# Upper limb movement quality measures: comparing IMUs and optical motion capture in stroke patients performing a drinking task

**DOI:** 10.3389/fdgth.2024.1359776

**Published:** 2024-03-28

**Authors:** T. Unger, R. de Sousa Ribeiro, M. Mokni, T. Weikert, J. Pohl, A. Schwarz, J.P.O. Held, L. Sauerzopf, B. Kühnis, E. Gavagnin, A.R. Luft, R. Gassert, O. Lambercy, C. Awai Easthope, J.G. Schönhammer

**Affiliations:** ^1^ DART Lab, Lake Lucerne Institute, Vitznau, Switzerland; ^2^ Rehabilitation Engineering Laboratory, ETH Zurich, Zurich, Switzerland; ^3^ Department of Neurology, UCLA, Los Angeles, CA, United States; ^4^ California Rehabilitation Institute, Los Angeles, CA, United States; ^5^ Ambulante Reha Triemli Zurich, Zurich, Switzerland; ^6^ ZHAW School of Health Sciences, Institute of Occupational Therapy, Winterthur, Switzerland; ^7^ Faculty of Medicine, University of Zurich, Zurich, Switzerland; ^8^ ZHAW School of Management and Law, Institute of Business Information Technology, Winterthur, Switzerland; ^9^ ZHAW School of Engineering, Centre for Artificial Intelligence, Winterthur, Switzerland; ^10^ Division of Vascular Neurology and Neurorehabilitation, Department of Neurology and Clinical Neuroscience Center, University of Zurich and University Hospital Zurich, Zurich, Switzerland; ^11^ cereneo, Center for Neurology and Rehabilitation, Vitznau, Switzerland

**Keywords:** measurement system comparison, IMU validation, neurorehabilitation after stroke, upper limb assessment, drinking task, movement quality, movement analisys

## Abstract

**Introduction:**

Clinical assessment of upper limb sensorimotor function post-stroke is often constrained by low sensitivity and limited information on movement quality. To address this gap, recent studies proposed a standardized instrumented drinking task, as a representative daily activity combining different components of functional arm use. Although kinematic movement quality measures for this task are well-established, and optical motion capture (OMC) has proven effective in their measurement, its clinical application remains limited. Inertial Measurement Units (IMUs) emerge as a promising low-cost and user-friendly alternative, yet their validity and clinical relevance compared to the gold standard OMC need investigation.

**Method:**

In this study, we conducted a measurement system comparison between IMUs and OMC, analyzing 15 established movement quality measures in 15 mild and moderate stroke patients performing the drinking task, using five IMUs placed on each wrist, upper arm, and trunk.

**Results:**

Our findings revealed strong agreement between the systems, with 12 out of 15 measures demonstrating clinical applicability, evidenced by Limits of Agreement (LoA) below the Minimum Clinically Important Differences (MCID) for each measure.

**Discussion:**

These results are promising, suggesting the clinical applicability of IMUs in quantifying movement quality for mildly and moderately impaired stroke patients performing the drinking task.

## 1 Introduction

The number of stroke incidents and the number of survivors are both increasing (1). Stroke is a major cause of disability (2) and leads to a growing number of stroke survivors with sensorimotor impairments. Up to 50% of these survivors experience chronic upper limb impairments (3, 4), affecting their independence and quality of life (5).

To meet the growing need for restoring upper limb sensorimotor function in stroke survivors, it is essential to maximize the effectiveness of rehabilitation strategies. Integral to this process are accurate and precise assessments. They are essential for both tailoring rehabilitation strategies and assessing the effectiveness of interventions. However, current clinical assessment strategies are primarily based on ordinal scales that rely on task completion evaluation or visual scoring by trained professionals, such as physiotherapists (6). This results in low sensitivity of the assessments and mostly fails to adequately quantify movement quality (7). Movement quality is defined by the similarity of patients’ movements in a motor task as compared to those of age-matched, able-bodied individuals (8). Assessing movement quality is important as it helps to distinguish between compensatory movements and true behavioral restitution (9, 7, 10).

There is a strong consensus on employing instrumented or technology-based assessments to overcome the existing limitations. Assessments based on movement kinematics can have higher sensitivity (11) and are optimally suited for quantifying movement quality (12). However, this approach brings with it two main challenges. First, movement kinematics need to be captured accurately and reliably. Second, measures that quantify movement quality with adequate clinimetric properties must be identified (13).

Previous research recommends using an instrumented drinking task to assess upper limb movement quality in mild and moderate stroke patients (8). This task comprises important movement primitives such as three-dimensional reaching and hand-to-mouth movement and additionally stands out through its ease of standardization. Kinematic measures, which quantify movement quality (14) and have good clinimetric properties (15–17), have been established for this task using Optical Motion Capture (OMC) (18). However, the cost and impracticality of OMC limit its translation into clinical application. This raises the question of whether there is a measurement system that is both accurate and precise enough to capture these established movement quality measures, while also being cost-effective and user-friendly.

Wearable Inertial Measurement Units (IMUs) are a promising candidate (19). Placed on multiple segments of the upper limb, they allow for a robust recomposition of arm kinematics, while remaining relatively low-cost. Additionally, IMUs have the potential to go beyond controlled environments and measure individuals during their daily life (20, 21). To evaluate the suitability of IMUs as an alternative to OMC for an instrumented drinking task, it is necessary to investigate the measurement agreement between IMUs and OMC. This evaluation should specifically focus on the measurement uncertainty of IMUs when determining the established movement quality measures in stroke patients with mild to moderate upper limb impairment. Three previous studies compared measurements from IMUs to OMC for the drinking task (22–24). However, none of these studies comprehensively investigated the full set of the mentioned movement quality measures among stroke patients.

With an eye toward clinical application, this underscores the need to validate the entire set of the proposed measures in the target group of stroke patients with moderate and mild upper limb impairments. Therefore, in this study, we conducted a measurement systems comparison between IMU and OMC, involving 15 stroke patients with moderate and mild upper limb impairments performing multiple trials of the drinking task. We reconstructed and compared kinematics, including angular kinematics and end-effector velocity, from IMUs and OMC. Using these data, we calculated and compared the full set of movement quality measures from IMUs and OMC. We then assessed the agreement between the systems for all proposed movement quality measures. Finally, we contextualized the measurement uncertainty of IMUs by determining whether the observed disagreements were within a clinically acceptable range for practical application.

We anticipated that the discrepancy between kinematics obtained from OMC and IMUs would be minor, showing small error and a strong linear relationship between angular kinematics and end-effector velocity. Therefore, we hypothesized that the measurement uncertainty of IMUs regarding the movement quality measures, as determined through comparison with OMC (gold standard), is below the clinically relevant change of measure. This would suggest that IMUs and OMC can be interchangeably used for the drinking task assessment without compromising the accuracy and precision of the movement quality measures. We tested the hypothesis by comparing the Limits of Agreement (*LoA*) from OMC and IMU for each movement quality measure to the minimal clinically important difference (*MCID*) of the respective measure.

## 2 Materials and methods

### 2.1 Study design and participants

This study was designed to evaluate the potential application of IMU sensors in quantifying upper limb movement quality in stroke patients. The focus was on analyzing well-established kinematic movement quality measures during the performance of a drinking task (see full list of measures in Supplementary Table S4) and validating these against OMC. The study adhered to the ethical guidelines set forth by the local ethics committee (BASEC-No: 2022-00491).

**Participants**. Stroke survivors were recruited from the University Hospital Zurich Stroke Registry and the cereneo clinic. Eligible participants were invited for a single-session measurement, lasting approximately two to three hours. Inclusion criteria mandated that participants be at least 18 years old, capable of providing informed consent, and have a confirmed diagnosis of stroke. Additionally, participants were required to have at least partial ability to perform a reaching movement, and to grasp a cup unassisted using a cylindrical grip with the affected hand. Exclusion criteria consisted of pre-existing upper limb deficits, such as orthopedic impairments, and other neurological conditions.

Key characteristics of interest in this study were gender, age, affected arm, time since stroke, dominant arm, and severity of upper limb motor impairment, which was quantified using the Fugl-Meyer Assessment for Upper Extremity (FMA-UE) (25). A trained evaluator performed the FMA-UE alongside the drinking task measurements in a single session.

The study included an ad-hoc sample size of 15 stroke patients [which is above previous sufficiently powered studies on IMU validation (26)], with a mean FMA-UE score of 54.6, indicating a moderate level of upper limb impairment (see Table 1).

**Table 1:** Demographic data and clinical characteristics of stroke patients.

P-ID	Months since stroke	Age	Gender	Dominant arm	Affected arm	FMA-UE
1	116	76	M	R	L	38
2	12	61	M	R	L	62
3	52	77	M	R	R	31
4	183	56	M	R	L	48
5	1	53	M	R	R	50
6	6	80	F	R	B	63
7	12	52	M	L	L	57
8	28	82	M	R	L	57
9	31	78	M	R	R	47
10	33	44	M	R	R	66
11	54	87	F	R	R	62
12	2	59	F	R	L	64
13	51	78	M	R	L	62
14	56	45	M	R	R	64
15	34	63	F	L	L	48
Mean	45	66.07	11M 4F	13R 2L	9L 5R 1B	54.6
SD	48.20	14.33				10.53

P-ID: Patient-ID; R: right; L: left ; B: both; M: male ; F: female; FMA-UE: Fugl-Meyer Assessment for Upper Extremity; SD: standard deviation.

**Figure 1:**
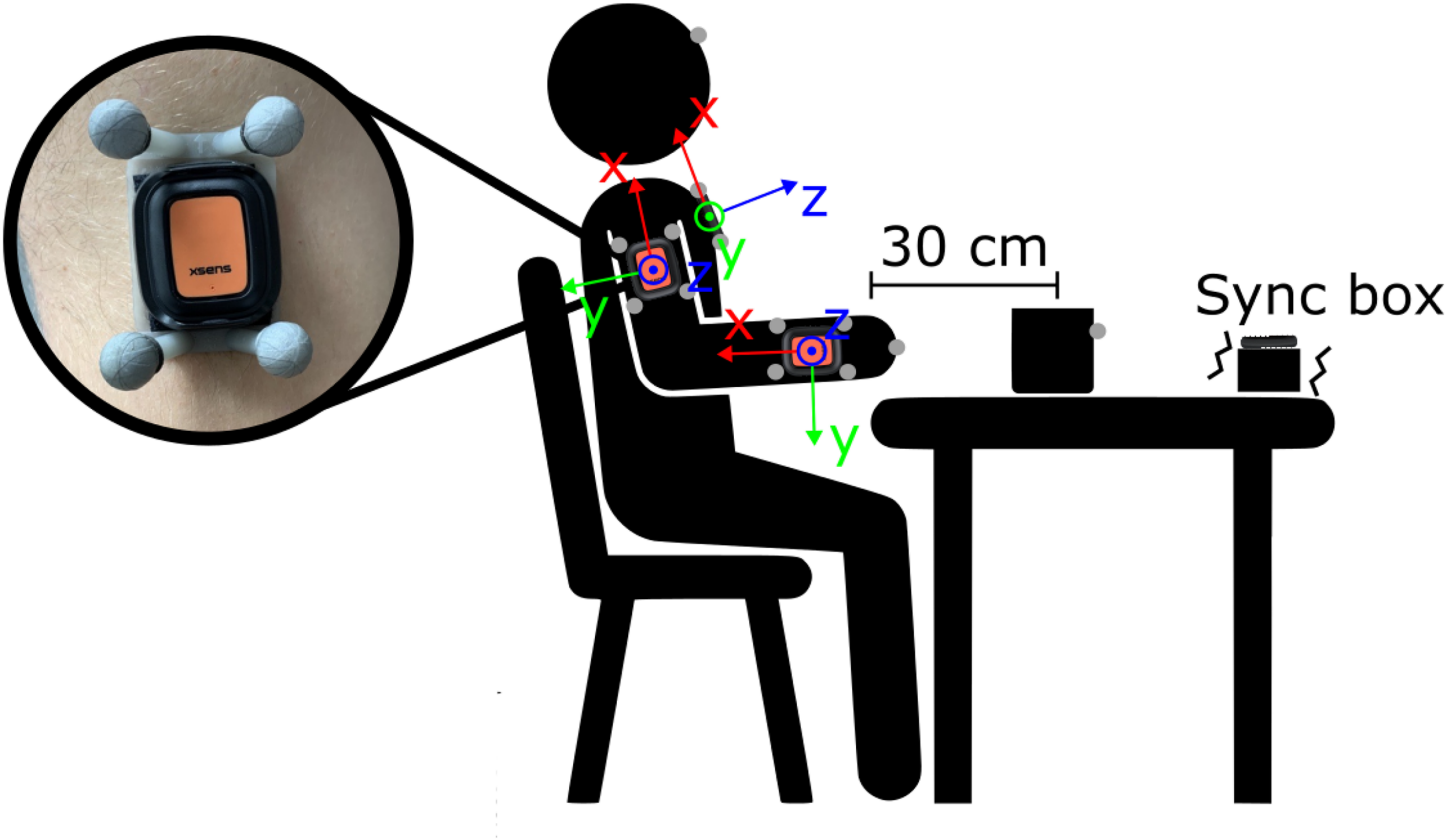
Sketch of measurement setup with the placement of IMUs, OMC clusters, and markers as well as the standardized sitting pose and positioning of the cup. Units of OMC clusters and IMUs share the same local coordinate system, as depicted in the figure.

**Measurement procedure**. The measurement procedure, adhering to established standards from prior studies (18), involved participants initiating and concluding a drinking task in a standardized pose. The cup was consistently positioned 30 cm from the table edge (see Figure 1). The cup was filled with approximately 100 ml of water and refilled between trials when necessary. Participants were instructed to take a sip of water on every trial. In the rare event that participants dropped the cup and spilled water (2 participants), the water was replaced with a ball of similar weight as the water. Participants were instructed to perform the drinking movement regardless. Participants completed 40 trials of the drinking task with the unaffected arm, followed by 40 trials with the affected arm. Each repetition of the drinking task is considered a trial. The unaffected arm was recorded as a proxy for control data of healthy, able-bodied, age-matched individuals. Each trial was individually recorded, with the OMC recording being started and stopped accordingly. The participants were verbally instructed when a trial started, and the recording was stopped once the trial was completed. Additional trials were carried out if any trials were suspected to be invalid (movement start before recording start, incomplete movement). After completion of the drinking task, the FMA-UE was conducted.

### 2.2 Measurement systems

This study utilized two primary measurement systems: IMUs and OMC.

**IMUs**. The IMU system utilized XSENS DOT units (27), recording offline at a rate of 120 Hz. The IMUs were internally synchronized with each other. Five IMUs were attached to the participants: one on each wrist and upper arm, and one on the trunk (see Figure 1). The IMUs were initially affixed to OMC clusters, then secured to the participants using a velcro band for the trunk and double-sided tape for the wrists and arms. The sensors were positioned to align with the anatomical axes of each body segment. Additionally, an IMU was mounted on a vibration box for synchronization, which vibrated as long as the OMC recording was ongoing.

**OMC**. The OMC system operated at a sampling frequency of 100 Hz, using technologies from Vicon, Qualisys, and Optitrack across different measurement setups. The five 3D-printed OMC clusters each featured a distinct configuration of four markers (see Figure 1). This design facilitated their automatic detection and labeling. In addition to the cluster markers, extra markers were placed on the middle knuckle of each hand, on the forehead and the cup, as required for movement phase classification in previous studies (18).

### 2.3 Data preprocessing

OMC data quality was high with few, mostly small gaps (<0.1 ms). All gaps were manually filled in Qualisys Track Manager using different methods depending on marker type (cluster markers: relational, within cluster rigid body; body markers: mostly polynomial or linear). Manual filling of gaps allowed for visual control of the validity of the filled gaps. Given the high quality of the raw data, along with careful manual filling and detailed visual inspection, we assume that the impact of filled gaps on the study results was negligible. Clean orientation data of clusters and positional data of markers were then exported to MATLAB. All further processing steps were done using custom-written MATLAB code.

IMU data was segmented into trials according to the vibration box’s synchronization signal. For the IMUs, two types of orientation estimation algorithms were utilized. First, we used XSENS DOT orientation estimation (DOT), employing accelerometer, gyroscope, and magnetometer data for 9D orientation estimation (27). The orientation estimation process occurs onboard the IMU, and the data is directly provided by the XSENS sensors. However, magnetometer data might be affected by ferromagnetic or electromagnetic interference in clinical contexts. Therefore, we also used the Versatile Quaternion-based Filter (VQF), which uses acceleration and gyroscope for 6D orientation estimation (28).

Both OMC and IMU data were then filtered using a 5 Hz low-pass Butterworth filter of fourth order. To align OMC and IMU data for direct comparison, the OMC data was resampled to match the IMU sampling frequency using spline interpolation. Upsampling was selected to ensure complete retention of information from IMU sampling points, which could be beneficial when calculating movement smoothness.

Initially, the orientation representations from the IMUs and OMC clusters do not share the same global coordinate system. To rectify this, the orientations of the OMC clusters at the start of each trial were used to rotate the global coordinate systems of each sensor into the global OMC coordinate system.

### 2.4 Kinematic trajectories

We first reconstructed kinematics trajectories, specifically the trajectories of angular kinematics and end-effector velocity. These kinematics form the basis for deriving most of the measures by, for example, taking peak values of angular kinematics or end-effector velocity during specific movement phases. Consequently, the accuracy of movement quality measures is inherently linked to the precision of these kinematics. Therefore, and to compare to other studies, we initially calculated kinematic trajectories and analyzed how IMU-based angular kinematics and end-effector velocities compare to those from OMC. Subsequently, we extracted movement quality measures and conducted a comprehensive measurement system comparison based on these. We first detail the calculation and analysis of the kinematic trajectories, and second the process for deriving movement quality measures and how we examined and contextualized their agreement between OMC and IMU.

The following kinematic trajectories are necessary for deriving all established movement quality measures for the drinking task (14, 18, 17, 29) (see Supplementary Table S4): *elbow angle*, *elbow angular velocity*, *shoulder angle (flexion / abduction)*, *trunk displacement angle* and *end-effector velocity*. In the following, the calculation of these kinematic trajectories is described. Since these calculations depend on orientation data, the same methods can be applied to both OMC and IMU, by utilizing orientation data from the OMC clusters and orientation data provided by the IMUs.

**End-effector velocity**. To determine the *end-effector velocity* based on orientation data, we employed a simple forward kinematic model driven by the orientation of the upper arm and the wrist (30, 31, 26). In this model, the shoulder is assumed to remain stationary. The kinematic model is described by the equation 1. The lengths of the upper and lower arms (both left and right) for each participant were determined by placing additional OMC markers on bony landmarks: the shoulder (RSHO for the right, LSHO for the left), elbow (RELL for the right, LELL for the left), and wrist (RWRM for the right, LWRM for the left), as detailed in (32). Segment lengths were determined by calculating the distances between these markers, specifically from the shoulder to the elbow for the upper arm, and from the elbow to the wrist for the lower arm. We then adjusted the kinematic model lengths accordingly. This model allows for the estimation of the end-effector position, which is temporally differentiated to calculate the *end-effector velocity*.

(1) $$T_{j}=R_{j}\left[\begin{array}{c} -l_{j}\\ 0\\ 0 \end{array}\right],\;H_{j}=\left[\begin{array}{cc} R_{j} & T_{j}\\ 0^{⊤} & 1 \end{array}\right],\;H_{\text{end-effector}}=H_{\text{upperarm}}H_{\text{\textbackslash{},forearm}}$$

Here, l describes the length of a segment, j indexes the segment (either upper arm or forearm), R describes the rotation matrix of the segment, T the translation vector, and H the transformation matrix.

Utilizing a kinematic model, driven by orientation enables the application of identical methods for both IMUs and OMC clusters for *end-effector velocity* estimation. This approach prevents discrepancies arising from different methods and facilitates direct comparison. Before comparing the *end-effector velocity* between IMU and OMC (system validation), we compared the model-based *end-effector velocity* from OMC clusters $V_{OMC}^{m}$ with the velocity of the hand marker $V_{OMC}^{h}$ to gain insights into the characteristics and validity of the kinematic model (model validation).

**Angular kinematics**. To calculate angular kinematics, the angle between two axes of interest was determined using Equation 2:

(2) $$\theta_{n}=\arccos\left(\frac{{v1}_{n}\cdot {v2}_{n}}{\left\|{v1}_{n}\|\|{v2}_{n}\right\|}\right)$$

Here, $\theta_{n}$ represents the angle at the $n^{th}$ sample. Vectors ${v1}_{n}$ and ${v2}_{n}$ correspond to the two axes of interest at the $n^{th}$ sample. Segment axes were derived from orientation data. For *elbow angle* calculation, we used the x-axes of the upper arm and wrist IMUs (see Figure 1). *Elbow angular velocity* is the derivative of the *elbow angle*.

Due to the natural curvature of participants’ chests, the trunk sensor often displayed an inclination, preventing it from showing a true vertical alignment in an upright seated position at the start of the measurement (see Figure 1). To address this, we performed a sensor-to-segment alignment by transforming the sensor orientation to match the trunk orientation. This adjustment was done at the beginning of each trial when patients were sitting upright in a standardized position. This method corrects for any initial inclination while maintaining the same directional heading. The resulting trunk orientation was used to calculate *trunk displacement angle*.

The vertical trunk axes alongside the x-axes of upper arm sensors were used to calculate the *shoulder angle*. To differentiate between *shoulder abduction* and *shoulder flexion*, the *shoulder angle* was projected onto the coronal and sagittal planes, respectively. The sagittal and coronal planes were defined based on the trunk orientation. Specifically, the coronal plane corresponds to the x-y-plane of the trunk, while the sagittal plane is determined by the x-z-plane (see Figure 1).

**Figure 2:**
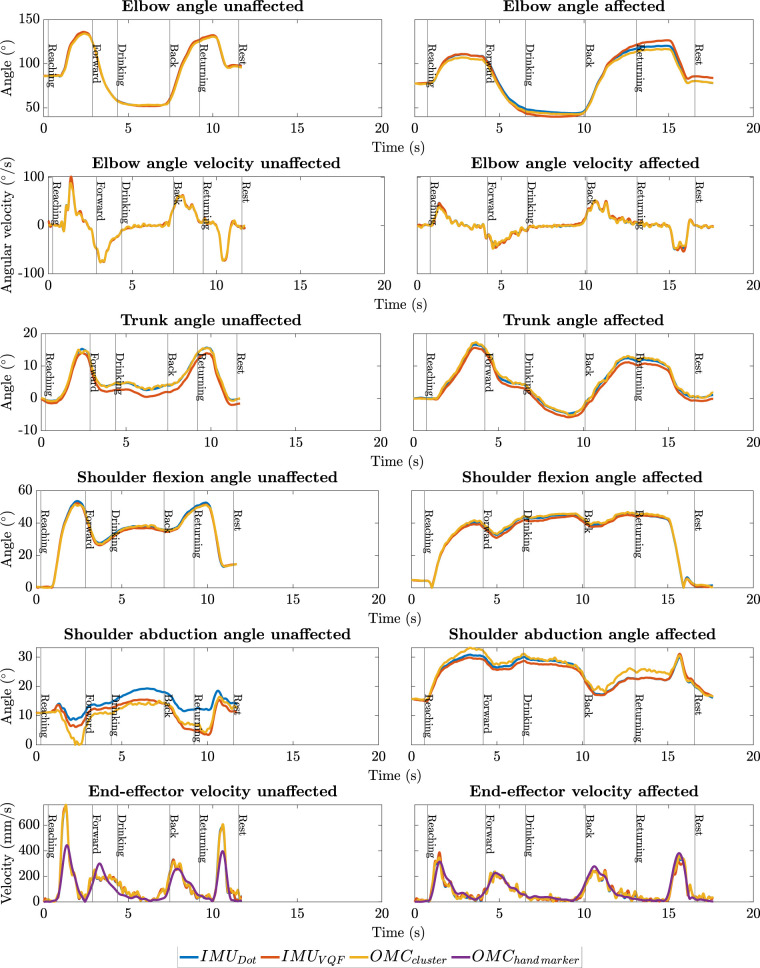
Exemplary kinematic trajectories of patient P9, showing one trial each for the affected and unaffected arm, and displaying all necessary kinematic trajectories for deriving the movement quality measures. Angular kinematics are presented using various methods including OMC cluster, VQF, and DOT. End-effector velocities are displayed for the kinematic model deploying orientation data from the OMC cluster, IMU (VQF and DOT), alongside hand marker velocity.

**Model validation**. Trajectories of the *end-effector velocity* based on the hand marker $V^{h}$ and the kinematic model $V^{m}$ were plotted for comparison (see Figure 2). Prior to utilizing the kinematic model with IMU and OMC orientation data to derive movement quality measures, we assessed the model’s validity by comparing the trajectories of *end-effector velocity* from the kinematic model $V_{OMC}^{m}$ with those from the hand marker $V_{OMC}^{h}$. This comparison employed measures such as root mean squared error *RMSE* and Pearson correlation *r* for the trajectory, along with characteristic values like differences in time to peak velocity (*Δt to PV*) and peak velocity (*ΔPV*) itself. These comparisons were aimed at evaluating the linear relationship and the magnitude of error between $V_{OMC}^{h}$ and $V_{OMC}^{m}$.

**System validation**. To assess the linear relationship and error in kinematic trajectories derived from IMU and OMC, we calculated *r* and *RMSE* for each kinematic trajectory. *RMSE* and *r* are frequently reported metrics in previous studies comparing kinematic trajectories from IMU and OMC. We reported the median and quartile ranges for *r* and *RMSE* across all trials, for both the affected and unaffected arm. For both model and system validation, the measures used for comparison were derived for each trial. The median and quartile range (first to last) across all trials are reported, distinguishing between the affected and unaffected arm. This distinction was made to assess whether pathological movement characteristics influence the validity of the kinematic model and the IMU system, respectively.

### 2.5 Movement quality measures

The movement quality measures included in this study are those previously established for the drinking task in various studies (14, 18). In this study, however, we shifted from measuring *trunk displacement* in [mm] to [∘]. This adjustment was made because IMUs demonstrated enhanced accuracy and precision in angle measurements compared to displacement metrics (33). We assume that compensatory trunk movements in a stationary, seated posture can be effectively quantified through both translational trunk displacement and the angular alteration of the trunk. Additionally, we investigated the acceleration-based Log Dimensionless Jerk (*LDLJ*) (34) as an additional measure for evaluating movement smoothness in the drinking task, alongside the *number of movement units* (see full list of measures in Supplementary Table S4). Unlike the *number of movement units*, this smoothness measure is independent of movement duration. Moreover, it is especially well-suited for measuring smoothness with IMUs, as the calculation is based on a derivative of the acceleration signal of the end-effector. Movement quality measures were derived from the kinematic trajectories and timestamps indicating the start and end of different phases of the drinking task (detailed in Table S4). These phases are: reaching, forward transport (moving the cup to the mouth), drinking, back transport (placing the cup back on the table, which includes releasing the grasp), and returning (moving the hand back to the starting position). The classification of these phases followed the same method used in previous studies (18) and results were used for both IMU and OMC-derived measures.

In the following, we explain the calculation of specific measures where understanding is not immediately evident from Table S4 and descriptions of kinematic trajectories. Straightforward measures, such as *peak velocity* during reaching, are not elaborated upon here.

The reaching movement starts at the first instance when the *end-effector velocity* exceeds 2% of its maximum. *Total movement time* is then defined as the duration between this starting point and the last instance when the *end-effector velocity* exceeds the 2% threshold.

The first peak velocity is identified as the first velocity peak in reaching exceeding a threshold of 10% of the *peak velocity*. The calculation of the *number of movement units* is based on the method from previous studies (18), involving the detection and counting of peaks that meet specific criteria in the *end-effector velocity* trajectory. The calculation of *LDLJ* (34) was performed using the acceleration signal from the IMU, and for OMC, it was done with acceleration obtained from the double differentiation of the hand marker’s position. *Interjoint coordination* is calculated with Pearson correlation between the *elbow angle* and *shoulder flexion* angle trajectory during the reaching phase.

### 2.6 Statistical analysis and clinical contextualization

**Measurement system comparison**. To visualize the agreement between OMC and IMUs in movement quality measures we plotted the results for each measure using both correlation and Bland–Altman plots. Furthermore, we averaged data points from all trials for each patient’s affected and unaffected arm into a single value for inclusion in these plots. This facilitates visual examination of correlation, bias, error variance, and heteroscedasticity.

We estimated *LOAs* using linear mixed-effects models since our data involved repeated measures within subjects (35). The *LOAs* for the differences between each OMC and IMU measure were calculated as the mean bias estimate plus or minus 1.96 times the standard deviation estimate.

For this purpose, the paired differences between each OMC and IMU measure were modeled with a mixed-effects model (random effect of patient and arm). The estimate of the standard deviation of the differences was obtained from the square root of the sum of the variance due to the effect of patient, due to the effect of trial, and the squared standard deviation of the residual.

Additionally, the difference between each OMC and IMU measure was modeled with a reduced mixed effects model (random effect of patient). The resulting intercept yields an appropriately weighted estimate of the mean bias (36).

We tested the reported correlation values for movement quality measures for statistical significance by applying a two-sided t-test, with a significance level set at p=.05.

**Clinical contextualization of measurement uncertainty**. In the context of introducing a new clinical measurement system, it is critical to assess the system’s measurement uncertainty. This can be achieved with *LOAs*, which define an interval that contains approximately 95% of measurement errors. *LOAs* can thus be considered a benchmark of tolerable measurement error (measurement uncertainty) of a system. The clinically tolerable amount of measurement uncertainty depends on the minimal change of interest in a given measure. For movement quality measures from the drinking task, this minimal change can be approximated by the measure’s Minimally Clinically Important Difference [*MCID*, (8)]. If each *LOA* is lower than the *MCID* for a measure, this suggests that the system is sensitive enough to detect all changes in the measure that are clinically important. Conversely, if a *LOA* exceeds the *MCID*, the measurement system may still be useful but is not able to capture changes in the measure down to the most granular level of sensitivity. Therefore, we used the *MCID* as a benchmark and reported whether the *LOA* is below or above the *MCID* for each measure.

**Estimation of *MCID*** Determining the *MCID* typically involves longitudinal studies and patient self-reports of improvement. This approach was applied in a previous study for two of the measures included here (8): *total movement time* and *number of movement units*. For the remaining measures, *MCIDs* do not exist and there is no established approach for their estimation from single time-point study data. It was proposed that a change in kinematic measures is clinically important if it is at least 15% of the range of a measure (8). Another approach is to estimate *MCIDs* as multiples of the standard deviation of a measure with the less impaired arm, analogously to studies with large normative data from an able-bodied, age-matched population (37). We calculate *MCIDs* for each measure using each of these approaches, and used the smallest resulting *MCID* as a benchmark, as a conservative option. A detailed explanation of the different methods and resulting *MCIDs* can be found in the Supplementary Material.

## 3 Results

### 3.1 Data set

In total 1127 trials of the drinking task of 15 patients were included with a median of 39 [35, 40] trials per arm per patient. Trials were excluded when the OMC recording did not include the entire movement, typically because the movement onset occurred before the recording started. Moreover, trials were excluded when the drinking task was not completed, such as when the cup was dropped. Finally, trials were excluded when OMC data were incomplete and could not be interpolated which was usually due to occluded markers.

### 3.2 Kinematics and movement quality measures

As mentioned above we first compared kinematic trajectories of OMC and IMU as well as *end-effector velocity* from the kinematic model and hand marker. Subsequently, we thoroughly analyzed the movement quality measures on which we ultimately judged the agreement between the systems.

### 3.3 Kinematic trajectories

Exemplary plots of reconstructed kinematic trajectories can be found in Figure 2. The in-depth analysis of kinematic trajectories described in the following and displayed in Table 2 compares IMU-based (DOT) kinematic trajectories with OMC-based kinematic trajectories as well as $V_{OMC}^{h}$ and $V_{OMC}^{m}$ (kinematic trajectories of IMUs based on VQF orientation, although also displayed in Figure 2, are not considered in this analysis).

**Table 2:** Medians and interquartile ranges for *r* and *RMSE* of kinematic trajectories were calculated across all patients, differentiating between the affected and unaffected arms.

Kinematics	Value	Unaffected arm	Affected arm
(a) Model validation $V_{OMC}^{h}$ vs. $V_{OMC}^{m}$			
*Velocity*	*r*	0.90 [0.87, 0.93]	0.90 [0.85, 0.92]
	*RMSE* (mm/s)	88.12 [76.05, 104.48]	94.72 [75.94, 118.81]
*Peak Velocity* ${\;}^{†}$	Δt to PV (s)	−0.03 [−0.11, 0.01]	−0.08 [−0.13, −0.02]
	ΔPV (mm/s)	62.66 [−90.70, 207.23]	122.87 [−66.39, 303.16]
(b) System validation $V_{OMC}^{m}$ vs. $V_{IMU}^{m}$			
*Velocity*	*r*	1.00 [0.99, 1.00]	0.99 [0.98, 1.00]
	*RMSE* (mm/s)	20.39 [14.27, 30.71]	20.64 [16.32, 29.04]
Angular kinematics			
*Elbow Angular Velocity*	*r*	0.99 [0.99, 0.99]	0.99 [0.98, 0.99]
	*RMSE* (°/s)	4.53 [3.11, 6.22]	4.61 [3.02, 6.89]
*Elbow Extension*	*r*	0.99 [0.99, 0.99]	0.99 [0.99, 0.99]
	*RMSE* (°)	2.78 [2.05, 4.02]	2.89 [2.07, 3.87]
*Shoulder Flexion*	*r*	0.99 [0.99, 0.99]	0.99 [0.99, 0.99]
	*RMSE* (°)	1.03 [0.71, 1.49]	1.05 [0.69, 1.51]
*Shoulder Abduction*	*r*	0.97 [0.94, 0.99]	0.98 [0.94, 0.99]
	*RMSE* (°)	2.15 [1.43, 3.63]	2.37 [1.37, 3.81]
*Trunk Angle*	*r*	0.99 [0.99, 0.99]	0.99 [0.98, 0.99]
	*RMSE* (°)	0.38 [0.27, 0.52]	0.49 [0.35, 0.71]

These measures were derived from the comparison of kinematic trajectories using IMU (DOT) and OMC (Cluster) for angular kinematics as well as hand marker and kinematic model for *end-effector velocity*. Besides correlation and *RMSE* between *end-effector velocity* trajectories, characteristic values of the *end-effector velocity* with the difference in time to peak velocity (Δt to PV) and the difference in peak velocity itself (ΔPV) are also reported (denoted with †). The upper indices m and h stand for m:model and h:hand marker.

**Model validation**. We observed a strong linear relationship between $V_{OMC}^{h}$ and $V_{OMC}^{m}$, demonstrated by a high median correlation (r>0.9). The median differences in time to peak velocity were minimal (unaffected arm: −0.03s; affected arm: −0.08s), confirming high temporal alignment in peak velocities.

While these results demonstrated good temporal alignment of $V_{OMC}^{h}$ and $V_{OMC}^{m}$, differences in the magnitude and smoothness of the signals were observed (see Figure 2). The median *RMSE* in velocity profiles was 94mm/s for the affected arm and 88 mm/s for the unaffected arm. ΔPV consistently exceeded the *RMSE*, reflecting greater variability at the peaks. This observation aligns with our visual inspection of the trajectories.

**System validation**. We observed a very high linear relationship for all kinematic trajectories between IMUs and OMC. This was reflected by high median correlations (r>0.99), with small interquartile ranges for all measurements. Intersystem correlation for shoulder abduction was slightly lower than for other kinematic trajectories with *r>0.97* (unaffected) and *r>0.98* (affected).

The *RMSEs* between OMC and IMUs were minimal for all kinematic trajectories. For the *end-effector velocity*, median *RMSEs* between the systems were fairly low with 20.39mm/s and 20.64mm/s for the unaffected and affected arm, respectively. The biggest *RMSEs* observed regarding the joint angles were in the elbow angle (unaffected: $2.7^{\circ }[2^{\circ },4^{\circ }]$, affected: $2.8^{\circ }[2^{\circ },3.8^{\circ }]$).

These findings demonstrate an overall high linear association and consistently low errors between the systems. Again, this observation was consistent with visual inspection of the trajectories.

### 3.4 Movement quality measures

**Figure 3:**
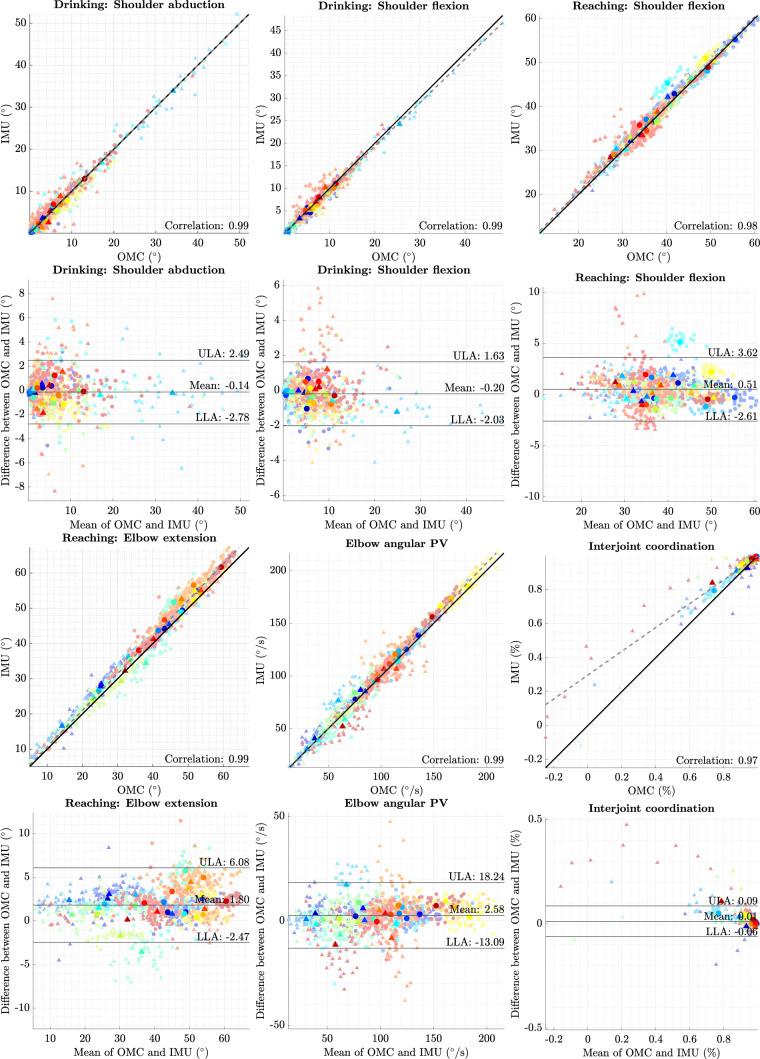

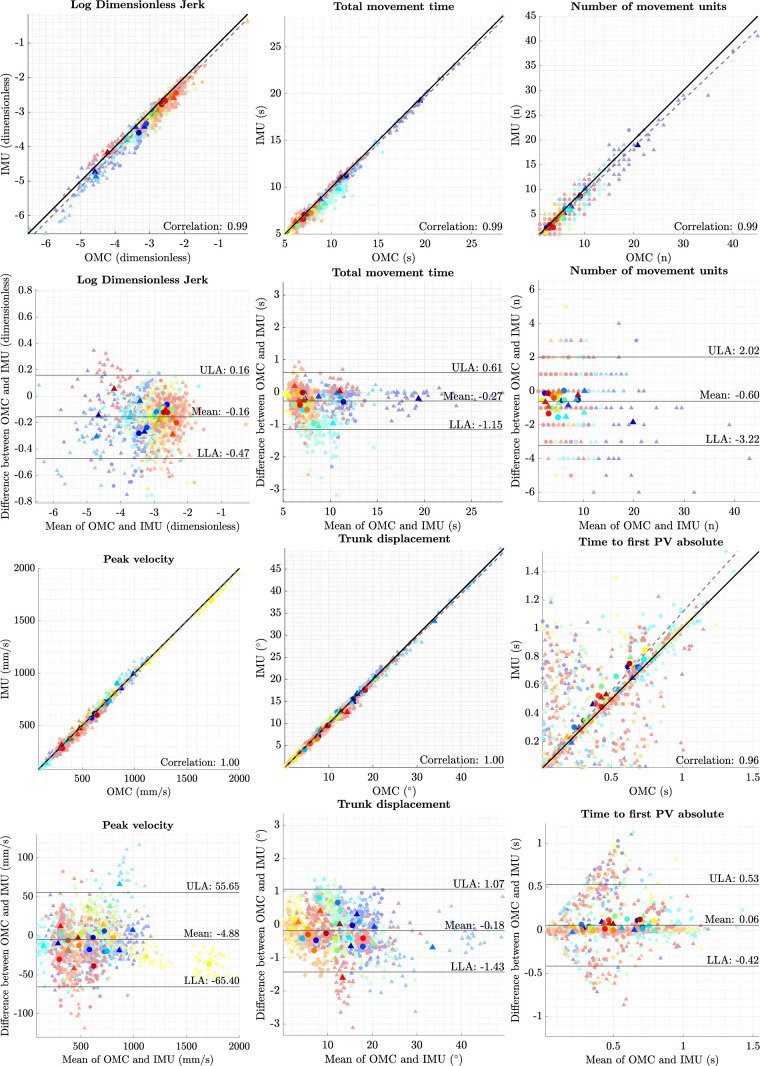

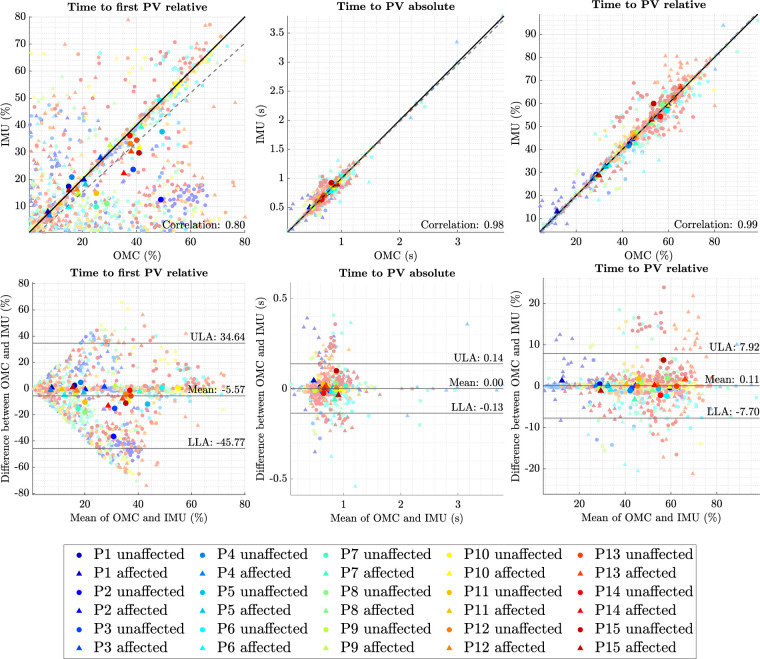
Correlation and Bland–Altman plots for each measure, comparing OMC and IMUs (DOT). Each data point with low opacity represents a single trial. Points with high opacity represent the average over a patient’s arm. Trials are color-coded per patient and shape-coded per arm. In the correlation plots, the Pearson correlation coefficient for average trials is denoted. The Deming regression line is displayed as a dashed grey line. In the Bland–Altman plots, bias and *LOAs* are displayed according to the results of mixed-effects model analysis.

The results for the movement quality measures of OMC and IMU (DOT) are shown in Figure 3 and summarized in Table 3. Results for VQF can be found in the Supplementary Material (see Table S7). In general, most of the measures showed high agreement between OMC and IMU, which can be visually observed in the plots (see Figure 3) and was supported by high Pearson correlations, narrow *LOAs* and low biases (see Table 3).

**Table 3:** Statistical summary and clinical contextualization of agreement between measures derived from OMC and IMUs (DOT): This table presents the Pearson correlations ($r_{single}$ and $r_{avrg}$) calculated across individual trials and averaged trials, respectively.

	Movement Quality Measure	$r_{single}$	$r_{avrg}$	*bias*	*ULA*	*LLA*	*MCID*	*LOA* < *MCID*
1	Velocity and movement time							
1.1	*Total movement time* [s]	0.99${\;}^{*}$	0.99${\;}^{*}$	−0.27	0.61	−1.15	2.4	y
1.2	*PV* [mm/s]	1${\;}^{*}$	1${\;}^{*}$	−4.88	54.96	−64.71	247.2	y
1.3	*Elbow angular PV* [°/s]	0.98${\;}^{*}$	0.99${\;}^{*}$	2.58	18.09	−12.94	29.5	y
2	Movement strategy							
2.1	*Time to PV* [s]	0.98${\;}^{*}$	0.98${\;}^{*}$	0	0.14	−0.13	0.4	y
2.2	*Time to first PV* [s]	0.7${\;}^{*}$	0.96${\;}^{*}$	0.06	0.53	−0.42	0.2	n
2.3	*Time to PV* [%]	0.97${\;}^{*}$	0.99${\;}^{*}$	0.11	7.92	−7.7	14.2	y
2.4	*Time to first PV* [%]	0.5${\;}^{*}$	0.8${\;}^{*}$	−5.57	34.3	−45.43	11.9	n
3	Smoothness and coordination of movement							
3.1	*Number of movement units* [n]	0.95${\;}^{*}$	0.99${\;}^{*}$	−0.6	2.01	−3.21	3	n
3.2	*Interjoint coordination*	0.96${\;}^{*}$	0.97${\;}^{*}$	1.2	8.5	−6.1	9.6	y
3.3	*Log Dimensionless Jerk*	0.97${\;}^{*}$	0.99${\;}^{*}$	−0.16	0.16	−0.47	0.7	y
4	Compensatory trunk displacement and maximal angular joint motions							
4.2	*Trunk displacement* [°]	1${\;}^{*}$	1${\;}^{*}$	−0.18	1.07	−1.43	7.4	y
4.3	*Shoulder flexion - R* [°]	0.98${\;}^{*}$	0.98${\;}^{*}$	0.51	3.61	−2.6	7.4	y
4.4	*Elbow extension - R* [°]	0.99${\;}^{*}$	0.99${\;}^{*}$	1.8	6.04	−2.43	8.7	y
4.5	*Shoulder abduction - D* [°]	0.98${\;}^{*}$	0.99${\;}^{*}$	−0.14	2.48	−2.77	7.4	y
4.6	*Shoulder flexion - D* [°]	0.99${\;}^{*}$	0.99${\;}^{*}$	−0.2	1.63	−2.03	6.7	y

*Bias* and *LOAs* are the results of mixed-effects model analysis. The *MCIDs* are those reported in Supplementary Table S6. The final column indicates whether the *LOAs* fall below the *MCID* thresholds (y: yes, n: no) ${\;}^{*}$(p<0.01).

*End-effector velocity*-based measures compared results from $V_{OMC}^{m}$ and $V_{IMU}^{m}$. Across the measures, we generally observed high correlations (0.96<r<1), accompanied by few and small outliers. An exception here is the *time to first peak velocity*, where several outliers in the single trials were observed, resulting in a funnel shape of error in Bland–Altman Plots and a lower correlation for averaged trials (r=0.8). Also, *interjoint coordination* showed a few outliers coming from two different patients.

### 3.5 Agreement of measures and clinical contextualization

Table 3 summarizes the results of comparing movement quality measures between OMC and IMU. It reports the Pearson correlation for both individual and averaged trials, as well as the bias and *LOAs* derived from the mixed-effects model. Additionally, the table reports the *MCIDs* to facilitate comparison with the upper (ULA) and lower (LLA) (*LOAs*). The last column evaluates whether the *LOAs* are smaller than the *MCIDs* (yes/no).

## 4 Discussion

### 4.1 Key summary

We hypothesized that IMUs are suitable for deriving established movement quality measures for the drinking task of stroke patients, with an agreement level high enough to satisfy clinical requirements when compared to the gold standard. We evaluated clinical relevance by checking whether the LOAs between OMC and IMU for each measure are below the *MCID* of the respective measure. We found that, except for *time to first peak velocity* and *number of movement units*, all measures fulfill the criteria.

### 4.2 Interpretation

**Kinematic trajectories**. The movement quality measures in our study were primarily based on *end-effector velocity* and angular kinematics. Therefore, we first validated our kinematic model for the calculation of *end-effector velocity* and compared the kinematic trajectories of IMU and OMC to gain insight into their agreement at a basic level of analysis.

We validated the kinematic model for estimating *end-effector velocity* by comparing it to the hand marker velocity. The high linear association (r>0.9) observed on both affected and unaffected arms, coupled with minimal temporal discrepancies in peak times, supports its temporal validity. However, some differences in peak velocity magnitudes between the model-based velocity and the hand marker velocity were noted, likely due to coarse precision in segment lengths, imperfect cluster/sensor-to-segment alignment, and the disregard of trunk-induced velocity. Moreover, the model-based velocity showed less smooth trajectories which might weaken its ability to accurately estimate the *number of movement units*. Our findings suggest that while peak velocities and smoothness may differ between these two methods, the kinematic model remains reliable for estimating *end-effector velocity*, particularly in temporal aspects. All kinematic trajectories exhibited a strong linear association across all trials for both affected and unaffected arms (medianr>0.97), along with a small median RMSE (*end-effector velocity*: 20mm/s, *joint angles*: $1^{\circ }$ to $3^{\circ }$) when comparing IMU and OMC measurements. Interestingly, there was no notable difference in RMSE and r between the affected and unaffected arms, indicating the robustness of IMUs in deriving kinematics even in the presence of pathological compensatory movements. The observed results for angular kinematics align with those reported in previous IMU validation studies (33).

It is important to note that tremors and small movement perturbations can impact the measurement accuracy of both IMU and OMC systems, and that these may be exacerbated by soft tissue effects (38, 39). Additionally, the orientation estimation of IMUs can drop in accuracy when tremor and small perturbations are present. This mainly becomes a problem for measurements over longer periods of time, where orientation estimation starts to drift (40). However, the measurement time for the drinking task is short and the magnitude of measurement error introduced by these factors is likely minor compared to the magnitude of the values of the established measures of the drinking task, which are focused on assessing gross upper limb movements.

***MCID***. We agreed that it is better to underestimate *MCIDs* than to overestimate them, and therefore applying stricter criteria regarding measurement uncertainty than may even be necessary. Before applying the *MCIDs*, we assessed whether our estimated *MCIDs* are likely to be valid and behave in such a way. We compared known *MCIDs* from previous studies (41) to those estimated by our approach.

These studies reported *MCIDs* ranging from 2.5 to 5 s for *total movement time* and 3 to 7 *movement units* for smoothness. In our study, the estimated *MCIDs* ranged from 2.4 s to 3.5 s for *total movement time*, and from 2.4 to 6.6 *movement units* for smoothness. Taking the lowest *MCID* as we did in this study consequently underestimated these known *MCIDs*. Given this, we assume that the *MCIDs* estimated for all other measures are valid, likely to fall within true ranges of *MCID*, with a tendency to underestimate them, and unlikely to overestimate them.

**Agreement of measures and clinical contextualization**. Visual inspection of Bland–Altman and correlation figures showed no difference in the agreement of IMU and OMC between the affected and unaffected arms. This is consistent with our previous analysis of kinematic trajectories. Consequently, we consider it valid to calculate the LOAs by pooling the data of both affected and unaffected arms.

The superior performance of measures calculated with DOT over those calculated with VQF may be attributed onboard orientation processing and access to a higher sample rate (800 Hz) of DOT (27). In contrast, VQF relies on raw sensor data of 120 Hz. In this study, the IMUs were used without performing prior intrinsic sensor hardware calibration (accelerometer, gyroscope, magnetometer) as intrinsic calibration methods are not available from the manufacturer. Since VQF is open source, fully transparent in processing, sensor brand independent, and offers orientation estimation both with and without a magnetometer it is still a considerable option for research and clinical application.

*LDLJ* was investigated alongside the recommended kinematic movement quality measures as an alternative or complementary measure of smoothness. It was previously validated for IMUs in a study involving four stroke patients performing tasks of the Action Research Arm Test (34). Our results demonstrate that IMU and OMC agree in measuring *LDLJ*, confirming these previous findings. Additionally, we expand upon the previous findings as by applying *LDLJ* to a different IMU sensor system (unlike the previously used Zurich Move IMUs), a larger population of stroke patients, and a different task, thereby suggesting broader applicability of the measure. The advantage of *LDLJ*, particularly its time independence, became apparent in Participant P1’s case. P1 required significantly more time to complete the drinking task with his affected arm compared to other participants, resulting in prolonged movement execution time and a consequential increase in movement units. However, when analyzed with *LDLJ*, P1’s movements showed no drastic deviation in *LDLJ* values compared to other patients like P3, who exhibited similar levels of impairment. We therefore suggest to add *LDLJ* as smoothness measures to the drinking task, especially when using IMUs.

Except for the *time to first peak velocity*, Bland–Altman and correlation plots showed no major outliers, with minor exceptions in *interjoint coordination*. These outliers primarily appeared in patients with higher levels of impairment and restricted joint mobility, particularly in *elbow angle* (extension) and *shoulder flexion*. As *interjoint coordination* is the correlation between *elbow angle* (extension) and *shoulder flexion*, the very limited range of motion in these joints makes the measure more susceptible to small inaccuracies. Consequently, even minor measurement errors can have a disproportionate impact on the calculation of *interjoint coordination*, underscoring the importance of precise measurement techniques in patients with limited joint mobility.

The lack of precision in the magnitude of *end-effector velocity* is also the primary cause of substantial noise in the estimation of *time to first peak velocity*, rendering these measures clinically unfit for the time being.

### 4.3 Comparison with other studies and generalizability of results

Research on the accuracy of kinematic measurements with IMUs often involve comparison to a gold standard. Technical studies often employ industrial robots as benchmark (42), but across studies the most commonly used gold standard are OMC systems. Studies on IMU accuracy investigated a wide range of body movements, from gait (43) to upper limb motions (33), and revealed that accuracy varies with both the type of movement and the specific joint or segment involved.

A recent detailed review of upper limb kinematic measurement with IMUs analyzed 52 validation studies and compared the data processing methods and measurement errors, specifically regarding upper limb joint angles (33). Across all studies, which used different sensors, algorithms and upper limb movement tasks, the discrepancy between IMU and OMC measurements for humerothoracic movements was around $4^{\circ }$. Specifically, the elbow joint measurements were found to be more accurate, with an RMSE as low as $2^{\circ }$. In contrast, the glenohumeral joint movements reported RMSEs of at least $6^{\circ }$, and the scapulothoracic joint movements had RMSEs exceeding $10^{\circ }$.

Our study did not examine some joint angles of the upper limb (e.g., internal rotation of shoulder, pronation/supination, scapulothoracic joint), since these were not required to derive the movement quality measures for the drinking task. However, the joint angles we did analyze aligned well with the existing literature, generally even showing better accuracy than previous studies with median RMSEs below $3^{\circ }$ for all included joint angles.

The results of our study also align with previous IMU validation studies specifically focusing on the drinking task in terms of joint angle accuracy (22, 24). In terms of end-effector velocity during the drinking task with IMUs our study is the first one to test a kinematic model for this purpose. While our method allows for very high temporal resolution and reconstruction of end-effector velocity, the integration-based analysis used in a previous study on the drinking task reports even smaller RMSEs (23), but such an analysis might be vulnerable to sensor drift. Thus, a combination of both methods would likely yield the most robust and accurate results. Our results suggest that the orientation estimation of Xsens IMUs surpasses other advanced algorithms (VQF), which is supported by other literature as well (33). This suggests that alternative IMU models might deliver slightly less precise orientation and kinematic measurements.

Furthermore, when considering the applicability of our methods to other movement tasks, existing research indicates that errors similar to those we reported for upper limb movements in the drinking task are likely to be observed (22, 33, 24, 23). Therefore, we anticipate our methodology will yield consistent error margins across various upper limb activities, assuming they do not significantly diverge from the movements studied in our drinking task research.

Our study stands out from other studies in that we not only compare the kinematic trajectories with each other but also the measures that can be used in clinical application, in this case, measures that quantify movement quality. Furthermore, we put the magnitude of measurement error in relation to a clinically acceptable error which increases the relevance of our methods and results for the translation into clinical application.

### 4.4 Limitations

Our analyses and results, while promising, do not yet provide the basis for a fully independent IMU-based solution for obtaining movement quality measures. The initial alignment of the global coordinate system between the OMC cluster and IMU also resolves potential heading differences between IMUs. The brief recording times minimized the impact of drift over time, which is a common issue with IMUs. Also, phase classification still purely relied on OMC data. Next, no segment-to-sensor calibrations were used, as we relied on sensor alignment with anatomical axes by careful placement. Additionally, we observed differences in velocity peak values between the employed kinematic model and hand marker velocity, which hinders cross-sensor system comparison of this value. Moreover, although the method we employed to calculate *MCIDs* from our study population’s data produces results consistent with those *MCIDs* that have been empirically studied, it is crucial to acknowledge that the majority of our *MCIDs* lack further empirical validation.

### 4.5 Implications and suggestions for future studies

Our study establishes a benchmark in IMU performance, demonstrating the feasibility of achieving a clinically acceptable level of agreement between OMC and IMU measurements to derive movement quality measures in stroke patients performing the drinking task. Our findings pave the way for future research aimed at developing a standalone IMU solution. The key areas for this future investigation include:

1. Standardization of sensor-segment alignment and placement: The development of a standardized protocol for sensor alignment and placement is essential for ensuring consistent and accurate data collection.
2. IMU phase classification: Implementing and validating an IMU phase classification algorithm is crucial for an IMU standalone solution.
3. Performance stability trials: Investigating the number of trials needed to average over to achieve performance stability with IMUs, similar to the established protocols for OMC (15), also is crucial for clinical application.
4. Empirical *MCIDs*: Further studies to derive *MCIDs* will strengthen both our findings and the validity of clinical contextualization.
5. Kinematic Model Enhancement: Enhancing the kinematic model, especially by incorporating more sophisticated methods for *end-effector velocity* calculation and adding kinematic constraints using tools like OpenSim/OpenSense, could significantly improve the reliability and robustness of the measurement system.

If future research successfully bridges these identified gaps enabling a standalone IMU solution with minimal error, our results point towards the clinical applicability of using IMU-instrumented drinking tasks to quantitatively assess upper limb movement quality in stroke patients.

## 5 Conclusion

In this study, we examined the agreement between OMC and IMUs in acquiring movement quality measures for stroke patients with mild to moderate impairment levels performing the drinking task. Our study goes beyond mere agreement analysis; it evaluates *LOAs* of each measure in relation to their respective *MCID*, thereby contextualizing the agreement in terms of its clinical relevance.

Our results indicate no notable differences in kinematic accuracy between groups of varying impairment levels, demonstrating the overall robustness of the IMUs in accurately capturing also pathological movements. All measures, except for the *time to first peak velocity* and *number of movement units*, exhibited *LOAs* below the *MCID*, indicating interchagebility of systems without relevant loss of precision. Additionally, We investigated LDLJ as an alternative measure of movement smoothness. Our findings both confirm and extend previous validation studies on LDLJ in terms of patient population, the performed tasks, a the specific sensors, suggesting its validity as obtained with IMUs accross sensors and tasks.

We recommend refining the calculation of *end-effector velocity* to reduce the impact of noise on the detection of the first peak velocity and differences in peak velocity when comparing hand marker velocities with kinematic model-based velocities.

The findings indicate the interchangeability of OMC and IMU systems without loss of precision for most measures, which is a promising development for their potential use in clinical settings to quantify movement quality. In conclusion, our study presents encouraging results that support the clinical validity and applicability of IMUs for the assessment of movement quality measures in stroke patients performing the drinking task.

## Data availability statement

The raw data supporting the conclusions of this article will be made available by the authors, without undue reservation.

## Ethics statement

The study involving humans was approved by Ethikkommission Nordwest- und Zentralschweiz. The study was conducted in accordance with the local legislation and institutional requirements. The participants provided their written informed consent to participate in this study.
